# Tobacco smoking patterns in the emirate of Abu Dhabi, United Arab Emirates: a comprehensive analysis of trends before and after the onset of the COVID-19 pandemic

**DOI:** 10.3389/fpubh.2025.1607965

**Published:** 2025-06-26

**Authors:** Zufishan Alam, Iffat Elbarazi, Budoor Al Shehhi, Maha Mohamed AlSafi, Mariam Al Wahedi, Ilfat Assaad Maarouf, Aamir Hassan, Mohammed Al-Houqani

**Affiliations:** ^1^School of Health and Environmental Studies, Hamdan Bin Mohammed Smart University, Dubai, United Arab Emirates; ^2^Institute of Public Health, College of Medicine and Health Sciences, United Arab Emirates University, Al Ain, United Arab Emirates; ^3^Abu Dhabi Public Health Centre, Abu Dhabi, United Arab Emirates; ^4^College of Medicine and Health Sciences, United Arab Emirates University, Al-Ain, United Arab Emirates

**Keywords:** COVID-19, Midwakh, smoking patterns, smoking prevalence rate, tobacco, United Arab Emirates

## Abstract

**Introduction:**

Tobacco smoking is a leading yet preventable risk factor for morbidity and mortality worldwide. As in western countries, the Arab world also encounters the growing tobacco epidemic. Assessment of smoking prevalence is a key step in identification of emerging trends, thus enabling planning of preventive measures.

**Methods:**

This study aimed to evaluate the prevalence of tobacco smoking among applicants of the Premarital Screening Program, in Abu Dhabi, United Arab Emirates during 2019–2023. Data was collected from all participants who applied for the program, through a questionnaire enquiring about tobacco smoking habits and methods of use. Overall smoking prevalence was assessed for the five-year period, as well as individually for each year. Smoking patterns were observed for three distinct COVID-19 periods: pre-COVID (2019), peri-COVID (2020–21) and post-COVID (2022–23). The smoking patterns were also examined by gender and nationality status. The findings are based on responses of 74791 applicants, of which 47% were females and 68.4% UAE nationals.

**Results:**

The reported overall tobacco smoking prevalence was 14.6%, with cigarette and Midwakh (handheld pipe) being the most commonly used methods (6.6%), followed by water pipe (2.2%). Smoking levels were reported to be significantly higher in men and UAE nationals compared to women and non-nationals respectively. A general decline was observed in point prevalence of tobacco smoking levels from the pre-COVID period (19.1%) to the peri-COVID period (15.1%), and further to the post-COVID period (13.5%) as the pandemic restrictions eased.

**Discussion:**

There is a need of control measures and interventions to maintain the positive behavioral change, brought by the COVID-19 that resulted in tobacco use reduction. Furthermore, steps to curb use of Midwakh, which is becoming an increasingly popular method of choice, are also required.

## 1 Introduction

Smoking is the proven cause of numerous health conditions including the wide variety of non-communicable diseases (1). Despite long run campaigns to raise awareness for smoking prevention, it remains a top priority worldwide, due to its detrimental effects on public health as well as the health of young adults (2). It is also considered as one of the key areas of intervention among top targeted health and lifestyle behaviors (1). Latest data depicting global trends reported nearly 8 million deaths as a result of smoking in 2019, including one in five male deaths (3). Moreover, prevalence of over 90% smokers to be addicted by the age of 25 has been reported, which is projected to increase in the near future (4, 5). Over 80% of the 1.3 billion tobacco users worldwide live in low- and middle-income countries, resulting in augmented burden of tobacco-related morbidity and mortality in the respective regions (2).

Tobacco use contributes to poverty by diverting household expenditure from basic needs such as food and shelter to tobacco (6). The direct healthcare costs associated with treating smoking-related diseases are also substantial, straining the healthcare resources (7). Additionally, smoking-related illnesses result in lost productivity, disability, and premature deaths, impacting societies and economies (2). The use of various tobacco products and their impact have been studied around the world, with research reporting an increase in inclination of people to use electronic nicotinic delivery system (ENDS) and heated tobacco products (8, 9). Literature suggests that possible reasons for people to prefer using ENDS include the misconceptions on reduced risk and harm, more acceptance in social events and community gatherings due to minimal odor and views that these products are nonaddictive and effective in smoking cessation (10). To combat the serious public health implications of smoking, governments and organizations around the world have implemented diverse tobacco control measures. These include public awareness campaigns, tobacco taxation, advertising restrictions, smoke-free policies, access to cessation services, and comprehensive tobacco control policies (8).

Moreover, evidence from research indicates that smoking initiation often occurs during adolescence, making it a critical concern for health of young adults (11). Young people who start smoking are more likely to become regular smokers and face a higher risk of developing smoking-related diseases later in life. The addictive nature of nicotine makes it challenging for young smokers to quit, leading to a lifelong dependence and continued health risks (12). Thus, imposing prevention programs that tend to deter young people from starting tobacco use and becoming addicted to tobacco products, could be the key solution to the problem. However, studies have reported that the success in reducing the prevalence of smoking tobacco use among young people is incomparable among different countries and additional efforts are required to improve these achievements (13). Highlighting and comparing prevalence among countries as well as identifying the attributive socio-cultural, economic and behavioral risk factors are keys to enhance these advancements (8).

In the Arab world tobacco consumption follows trends similar to that in other regions of the world, however, the availability of different traditional, as well as the new and emerging methods have aggravated the issue and posed greater challenges (14). The emerging upward trend in youth tobacco use is a growing concern in the Middle East and North Africa (MENA) region. Recent studies carried out among university students in United Arab Emirates, Sudan and Saudi Arabia reported the smoking prevalence to be 15.1, 48.8 and 31.3%, respectively (15–17). Additionally, poorly established guidelines and policies in many Arab countries, have led to the increased utilization of different tobacco products, with a clear escalation in waterpipe use and vaping (8, 14). Thus, these emerging tendencies have tremendously contributed to the complex tobacco epidemic in the region.

Tobacco control in the Arab world faces common challenges and patterns that are influenced by shared cultural and socio-political features. Furthermore, it is compounded by forces that promote tobacco use, such as the tobacco industry (18). Based on available data from Arab countries, three major trends stand out including: the high prevalence of cigarette smoking among Arab men compared to women although there is an increase among females especially among young women; the resurgence of waterpipe smoking as a popular tobacco use method, particularly among the young adults; and the inadequate capacity of policies to effectively combat the tobacco epidemic (14). An important step that can help curb the epidemic impact in this region, is initiating population-based surveillance systems. In fact, Arab countries and health departments are short in population-based surveillance systems that help monitor the tobacco epidemic and prioritize actions and programs.

Within the United Arab Emirates, few studies have been carried out on the assessment of smoking prevalence among the general population. A recent systematic review summarized studies on tobacco consumption practices and interventions in the UAE, of which five reported the smoking prevalence in different regions of the UAE including Abu Dhabi, Dubai and Ajman (19). Of the reviewed articles, only two studies addressed large sample sizes, reporting smoking prevalence during 2008–2010 and 2011 in the Abu Dhabi Emirate, respectively (20, 21). The remaining three studies assessed prevalence in specific populations with small sample sizes, such as in secondary school children and individuals enrolled in the rehabilitation center or by specific modes of tobacco smoking (22–24). Hence, there is dearth of research on recent trends in tobacco smoking in the UAE. With the outbreak of COVID-19 pandemic, numerous challenges in the control of tobacco use came to surface, as the smoking patterns were noticeably affected. Therefore, it became even more crucial to study different smoking patterns prior to and following the onset of pandemic. Thus, this study aimed to assess the prevalence of tobacco smoking among adult population of the United Arab Emirates residing in Abu Dhabi Emirate from 2019 to 2023, respectively.

## 2 Methods

### 2.1 Study design and data source

This retrospective cross-sectional study utilized the Premarital Screening data reported to the Abu Dhabi Public Health Center (ADPHC) and the Department of Health (DOH). It aimed to estimate prevalence of tobacco smoking and its modes (cigarette, Midwakh and water pipe) among the adult UAE population taking part in the premarital screening program. The data is based on information acquired via surveys and clinical tests, mandated by law, to be provided by the individuals. Applying for a marriage certificate in Abu Dhabi, UAE. The tests screen common genetic conditions and infectious diseases whereas the questionnaire enquires into a variety of health behavioral risk factors, including tobacco use. Ethical approval for the study was obtained from Abu Dhabi Health Research and Technology Ethics Committee with reference: DOH/CVDC/2022/907. Requirement for consent for this study was waived by the ethics committee, as it was a retrospective study of the information collected via surveys for the premarital screening program, with authors having no access to personal information of the participants

### 2.2 Participants

For the purpose of this study, data collected during 2019–2023 was utilized. The population of interest comprised adults aged 18 years and above, irrespective of gender and nationality and residing in Abu Dhabi. All applicants taking part in the premarital screening program to apply for the marriage license during the study period were included, thus presenting a census of marriage license applicants.

### 2.3 Data collection

The questionnaire enquired on the participants’ demographics including gender, age and nationality. Their tobacco smoking status (if they smoke currently or not), and the mode of smoking used (cigarette, Midwakh or waterpipe). The tool has been previously used in a similar study (21). In the present study, current smokers were defined as individuals who either reported having smoked at least one cigarette per day (regular smokers) or reported smoking on some days (occasional smokers). Midwakh (handheld pipe) smokers were defined as applicants who reported having smoked at least one Midwakh per day or on some days. Whereas waterpipe smokers were defined as individuals who smoked at least one waterpipe head per week or those who smoked regularly. The individuals who reported that they had stopped all forms of smoking for at least last 6 months were considered ex-smokers.

### 2.4 Statistical analysis

Data was analyzed Microsoft Excel 365. Descriptive statistics including frequencies and percentages were used to summarize the demographic characteristics and smoking behavior of the participants. The main outcome measure was the overall prevalence of tobacco smoking during the stated period (2019–2023) and annual point prevalence during each of the respective years separately. The overall period prevalence for smoking was calculated by dividing the number of applicants reporting smoking from 2019 to 2023 by the total number of applicants applying for premarital screening program during the overall period. The estimated annual point prevalence was calculated by dividing the number of applicants who reported smoking by the total number of applicants applying that year.

For the purpose of analysis, ex-smokers were coded as non-smokers. Smoking status was examined by gender (male, female) and nationality (Emirati, non-Emirati). To assess the patterns brought about by the pandemic, the year 2019 was called the “pre-COVID” period, representing the time before pandemic started. 2020 and 2021 were grouped as “peri-COVID” period, which covered the main years when the pandemic was at its peak. 2022 and 2023 were labeled as the “post-COVID” period, indicating the time after pandemic, when situation started returning to normal. Chi squared tests were carried out to examine statistically significant associations between smoking patterns and gender, nationality and COVID-19 time periods. Additionally, correlation analysis was conducted to assess the relationship between smoking and age. Temporal trends in tobacco smoking prevalence were visualized using line and bar charts showing annual prevalence from 2019 to 2023 by different modes of smoking as well as demographic characteristics such as gender and nationality. As the missing data was less (3.2%) complete cases analysis was used, where records with incomplete data were excluded from analysis as appropriate.

## 3 Results

A total of 74,791 applicants, aged 18 years and above, registered in the premarital screening program, were included in the analysis. Table 1 summarizes the demographic characteristics of the study sample. Of the included participants, almost half (53%) were males and the majority (68.4%) of the participants were UAE nationals. The mean age of the included participants was reported as 25.50 years (S. D ± 2.12) with the majority of the individuals being within the age range of 18–29 years (58.9%).

**Table 1:** Demographic characteristics of participants from Abu Dhabi premarital screening program 2019–2023 (*n* = 74,791).

Demographic characteristics	Frequency (N)	Percentage (%)
Gender		
Female	35,137	47.0
Male	39,654	53.0
Total	74,791	100
Nationality		
UAE (National)	51,166	68.4
Expatriate	23,625	31.6
Total	74,791	100
Age Group		
18–29	44,076	58.9
30–39	21,459	28.7
40–49	6,783	9.1
50–59	1763	2.4
60 and above	710	0.9
Total	74,791	100
Self-reported smoking status among participants		
No	61,425	82.1
Yes	10,945	14.6
Missing	2,421	3.2
Total	74,791	100
Type of Tobacco smoking*		
Cigarette	4,944	6.6
Midwakh	4,913	6.6
Waterpipe	1,678	2.2

*Individuals smoking more than one tobacco type, reported more than once.

### 3.1 Prevalence of tobacco use and modes

Overall, for the study period 2019–2023, the prevalence of current tobacco smoking was reported to be 14.6%, with 6.6% of the overall participants smoking cigarettes, 6.6% Midwakh and 2.2% waterpipe, respectively (Table 1). Smoking prevalence was significantly higher in men (26.0%) than women (1.9%) and in nationals (15.6%) than non-nationals (12.6%; Table 2). Men were more likely (11.5%) than women (1.1%) to be cigarette smokers. Similar trend was observed for Midwakh use, with women being less likely to smoke Midwakh (0.1%) than men (12.3%; Table 3). The overall prevalence of waterpipe smoking was comparatively less than the other two modes, with 3.5% in males and 0.8 % in females (Table 3; Supplementary Figure 2). Cigarette (15.6%) and waterpipe smoking (3.8%) was found to be higher among non-Emirati men whereas the male UAE nationals had the highest Midwakh smoking prevalence (15.4%; Table 3).

**Table 2:** Overall smoking prevalence in 74,791 participants by gender and nationality from 2019 to 23.

Variable	Smoking status			Chi square test
	Category	Smokers N (%)	Non-smokers N (%)	
Gender	Female	654 (1.9)	33,526 (95.4)	χ^2^ = 8,654.67; df = 1; *p* < 0.001
	Male	10,291 (26.0)	27,899 (70.4)	
Nationality	UAE Nationals	7,962 (15.6)	41,511 (81.1)	χ^2^ = 111.42; df = 1; *p* < 0.001
	Non-Nationals	2,983 (12.6)	19,914 (38.9)	

**Table 3:** Overall self-reported smoking prevalence among 74,791 participants, by type of tobacco product based on gender and nationality status (2019–23).

Type tobacco	National *N (%)*		Expatriate *N (%)*		Overall *N (%)*		
	Female	Male	Female	Male	Female	Male	Overall
Cigarette	35 (0.2)	2,868 (10.0)	334 (2.6)	1,697 (15.6)	369 (1.1)	4,565 (11.5)	4,934 (6.6)
Midwakh	25 (0.1)	4,448 (15.4)	21 (0.2)	410 (3.8)	46 (0.1)	4,858 (12.3)	4,904 (6.6)
Waterpipe	48 (0.2)	989 (3.4)	222 (1.7)	412 (3.8)	270 (0.8)	1,401 (3.5)	1,671 (2.2)
Overall	108 (0.5)	8,305 (29.4)	577 (4.5)	2,519 (23.2)	685 (1.9)	10,824 (27.3)	11,509 (15.4)

**Figure 1:**
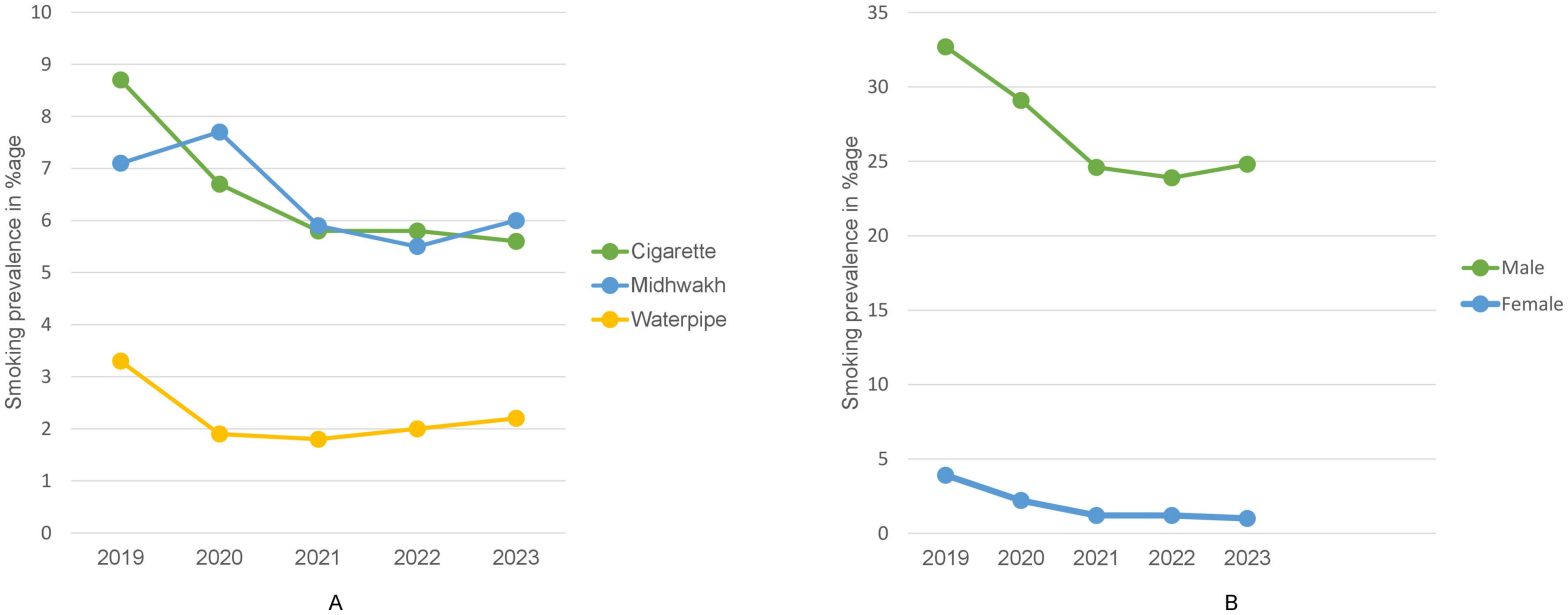
Trends in tobacco smoking prevalence 2019–2023. **(A)** Trend in point prevalence of tobacco smoking from 2019 to 2023 among the overall applicants of Premarital Screening Program, Abu Dhabi based on smoking type. **(B)** Annual trend in point prevalence of tobacco smoking from 2019 to 2023 among the male and female applicants of premarital screening program, Abu Dhabi.

The annual point prevalence for tobacco use was found to range from 19.1% (in 2019) before the onset of COVID-19 pandemic to 13.8% (in 2023) after the COVID-19 pandemic ended (Figure 1A). Highest point prevalence (19.1%) was observed during 2019, pre-COVID with 32.7% among males and 3.9% in females. Whereas lowest rate (13.2%) was reported for 2022, post COVID-19 when the restrictions for COVID-19 were relaxed, with 23.9% among males and 1.2% in females (Figure 1B; Supplementary Table 1). A statistically significant drop was therefore observed in tobacco smoking rates when pre-, peri-and post-COVID-19 time periods were compared (χ^2^ = 235.41, df = 2, *p* < 0.001; Table 4). During the pandemic, the drop in smoking levels was observed for both nationals as well as expatriates, yet it was higher in the latter group than the former. With regards to point prevalence for specific methods among overall participants varied patterns were observed, cigarette was the most commonly used method during 2019 and 2022, i.e., before COVID-19 started and in the first year as it progressed, whereas Midwakh was most widely utilized during 2020, 2021 (peri-COVID-19) period as well as in 2023. It appears that Midwakh use was the preferred method of choice during peri-COVID-19 times compared to cigarettes (2020–2021) with cigarette use resurging again post-COVID-19 in 2022, but Midwakh still being popular. However, in 2023, Midwakh was observed to be more widely utilized. In the overall population, water pipe was the least common method used each year compared to the other two modalities, ranging from 5% pre-COVID-19 (2019) to 3.3% (2022). But it is notable that post COVID-19, during 2023, use of waterpipe increased again (3.5%; Supplementary Table 1; Figure 1A).

**Table 4:** Smoking prevalence in 74,791 participants by the three COVID-19 periods.

COVID-19 period	Years	Smokers *N (%)*	Non-smokers *N (%)*	Chi square test
Pre-COVID	2019	2,933 (19.1)	12,041 (80.9)	χ^2^ = 235.41; df = 2; *p* < 0.001
Peri-COVID	2020–21	5,198 (15.1)	28,476 (84.9)	
Post-COVID	2022–23	3,378 (13.5)	20,908 (86.5)	

**Figure 2:**
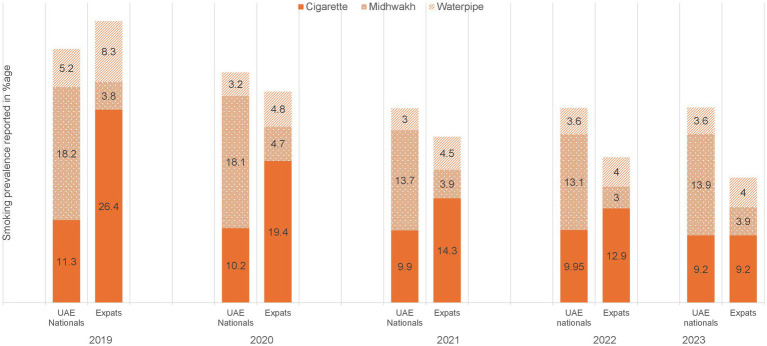
Annual trend in point prevalence of modes of tobacco smoking by nationality from 2019 to 2023 among applicants of premarital screening program, Abu Dhabi.

Differences were also observed in annual prevalence among subgroups. When observed based on gender, more males were found to use tobacco products than females across all 5 years (Figure 1B). Based on nationality, overall smoking prevalence was higher among national males as compared to expats, however it was opposite for females where expat women used smoking products more commonly as compared to nationals, throughout each of the 5 years. Interestingly, point prevalence was reported to be higher in expatriates than the nationals pre-COVID followed by decline and lower rates than nationals for peri- and post-COVID periods. (Supplementary Table 1; Figure 2).

Additionally, differences in the choice of modes were also observed based on gender and nationality. Both national and non-national females reported using cigarettes more often across each of the 5 years, followed by water pipe, whereas Midwakh was least commonly preferred method for them (Supplementary Table 1). In males, Midwakh was the most commonly chosen modality during peri-COVID 19 times and in 2023, whereas cigarette smoking was more popular choice during pre-COVID 19 and during 2022 (Supplementary Figure 3). Midwakh was the preferred choice for nationals while expatriates smoked cigarettes more commonly. Waterpipe was the least common choice for nationals during the 5 years (2019–2023). However, waterpipe was found to be the second most popular option among expats as compared to UAE nationals (Supplementary Figure 3; Supplementary Table 1). Correlation analysis between smoking and age revealed a weak but statistically significant positive relationship (*r* = 0.0607, *p* < 0.001).

## 4 Discussion

This study reports smoking prevalence and preferred methods of tobacco use among the adult UAE population before, during and after the onset of COVID-19 pandemic (2019–2023). The overall self-reported smoking prevalence was reported as 14.6% whereas point prevalence 19.1, 15.1 and 13.5% for the pre-, peri and post-COVID time periods, respectively. Prior to the onset of pandemic, cigarette smoking was the most commonly used option for the overall applicants, however it was replaced by Midwakh during peri-COVID period and continued to remain so post-COVID. The study also outlines the overall tobacco smoking prevalence trends by gender, with males having significantly higher smoking prevalence as compared to females. Similarly, UAE nationals had higher overall smoking prevalence compared to non-nationals.

The prevalence reported in the current study, when compared to earlier studies demonstrate an increase in the smoking prevalence. Specifically, when contrasted to a similar study utilizing Premarital Screening Program data during 2011 in Abu Dhabi, UAE, the former study reported prevalence of 24.7% among UAE national males that was lower than prevalence among the group (26.0%) reported in the current study (21). Two other studies assessing prevalence of current tobacco smoking among secondary school children in Dubai (2014) and Ajman (2015), reported it to be 23.4 and 24%, respectively (22, 23). Another cohort study carried out in 2016, comparing male individuals suffering from substance abuse disorder attending the National Rehabilitation Center, Abu Dhabi with general male population indicated it to be 95.6% versus 19.7%, respectively (24). However, another more comprehensive study reporting smoking prevalence from a large sample size, recruited via Weqaya Screening program (a program that screens individuals for risk factors for non-communicable diseases) for the years 2008–2010, reported overall prevalence (11.0%) to be lower than in the current study (14.6%), but it is notable that the referred study included UAE nationals as participants only (19). Similarly, a recent online survey-based study reporting smoking prevalence among UAE university students indicated it to be 15.1%, comparable to the results from our study (15). Thus, it appears that overall smoking levels have slightly increased compared to what was reported in the past and continues to remain high in population subgroups such as Emirati males. It is noteworthy that UAE has been included in the list of highest achieving countries in 2020 by WHO, in terms of enforcing bans on tobacco smoking advertisement, sponsorship and promotion as well as tobacco dependence treatment (6) and implementation of smoking control measures during the last 10 years in the region and in Abu Dhabi Emirates (25). Nevertheless, more effort is required, sustaining the improvement of prevention efforts and introducing new and effective programs.

Several studies around the world have cross sectionally examined smoking prevalence across the series of years but few have addressed the changes before, during and after COVID-19 era, with none in the Middle eastern region specifically. A significant drop was observed in point prevalence across the COVID-19 time period in our study ranging from 19.1% (pre-COVID) to 15.1% (peri-COVID) and 13.5% (post-COVID). This finding reflects a decrease in smoking levels possibly due to the lack of access to products secondary to lockdown restrictions, as well as information dissemination efforts linking tobacco use with worst outcomes for COVID-19 patients (8). Studies from other countries have reported mixed results for smoking prevalence during COVID-19 (8). A study investigating changes in tobacco smoking among young adults during lockdown period in Denmark, reported that for majority of participants smoking patterns did not change, but for one fourth it increased, with decrease in one fourth (26). However, few other studies conducted in Italy, U.S. and Ireland reported opposite suggesting increase in smoking (27–29). Changes in tobacco consumption behaviors during peri-COVID period have been studied in Arab countries as well. For example, a study reporting the impact of COVID-19 lockdown on smoking habits in 10 Arab countries in 2020 found that the overall smoking rate lowered. (30). Research has also been carried out to investigate the smoking trends among vulnerable populations during peri-COVID times. A US study including subgroups that were socioeconomically disadvantaged, suffering from substance abuse problems and mental health issues, reported that for them tobacco smoking increased, and they left homes to purchase cigarettes despite lockdowns (31). Future research needs to focus on examining smoking patterns among underprivileged and at-risk populations. Although overall smoking prevalence for post-COVID period remained low (13.5%) yet an interesting finding was a minor surge in smoking from 13.2% in 2022 to 13.8% in 2023. Therefore, it is crucial to take steps before the levels rise to pre-COVID levels in the coming years.

The results from the study suggest that cigarette was the preferred method of use pre-COVID, with Midwakh being the most commonly used method during peri-COVID times. After COVID-19 Midwakh and cigarette remained equally popular in 2022, whereas in 2023, Midwakh became the preferred option again. In contrast, water pipe was the least commonly used method. The pattern regarding use of water pipe as least common option, is optimistic, and is lower when compared to waterpipe smoking in other Arab countries such as Qatar, where it was reported to be 25.2% in 2019 (32). A possible reason for the low waterpipe utilization rates in UAE could be the ban that was introduced among 15 countries of Eastern Mediterranean region including UAE (8). In addition, the implementation of lockdown measures, imposed limitations on public access to waterpipes, and the restrictions on visits and movement also constrained the use of waterpipe within homes, which is commonly practiced as social activity (32, 33). However, the trend that Midwakh is becoming increasingly popular especially among national males and was used even more than cigarette during the peak COVID-19 period, is alarming. Increased utilization of Midwakh was also reported in a previous study, indicating Midwakh gaining more popularity among the UAE population (20). It implies need for urgency of steps required to regulate the use of such heated tobacco products.

Another observation from our study, indicating that males were significantly more likely to smoke tobacco than females, is consistent with previous studies (19, 20). The prevalence among males (41.9%) versus females (7%) reported in the previous Premarital Screening data-based study (2011) has found to have decreased in current study. It was also observed in our study that smoking prevalence for all three modes was higher among expat females as compared to national females. This is in agreement with what was previously reported by Aden et al. (21). The smoking prevalence for all three modes decreased from 2019 to 2023 for both national and expat males. Our study indicated that there was a decrease in smoking levels in both males and females throughout the 4 years after pre-COVID, whereas other studies in different geographical regions for instance in Italy, have reported that females tended to smoke more than males during the peri-COVID time (27). It is notable that our study captured self-reported smoking uptake across the three main burned tobacco products but did not include ENDS. A possible explanation of the decrease in the smoking prevalence during 2020–2023 as seen in our study could be attributed to the increased popularity and use of ENDS during the COVID-19 period. This could also hold true for females contributing to decrease in respective smoking prevalence, secondary to their preference to use ENDs compared to the traditional tobacco products.

Literature appraisal for tobacco smoking point prevalence levels in other countries of the Arab region suggests that it is still high in a few countries. The reported levels include 39.2% during 2014 in Kuwait, 21.3% during 2018 in Saudi Arabia, 25.2% during 2019 in Qatar and 51.1% during 2021 in Jordan (32, 34–36) respectively. Although UAE appears to be on appropriate track in lowering smoking levels, more efforts are required to incorporate most effective policies recommended by MPOWER (8) not only to restrain tobacco use within the region but also to sustain existing low prevalence levels. Specific attention needs to be paid to monitor and curb the use of ENDS, as UAE has not been able to implement any ban on the respective products (8).

### 4.1 Limitations

This study reported findings based on data retrieved from a large sample size-based survey and to our knowledge is the first to report varying trends in prevalence brought by COVID-19 pandemic in any of the middle eastern countries. Being a program that targets mainly young adults, Premarital Screening Program is an effective medium to gain data based on insight from youth practices. It also serves as valuable source for possible implications for long-term impact on health, socio-economic and cultural factors. However, there can be a potential of recall and social desirability bias, as the data was self-reported. The data was collected from applicants of the Premarital Screening program in Abu Dhabi; therefore, it cannot be generalized to the overall population subgroups. However, this in one way can be considered as a strength as it may provide an idea on the smoking behavior of young adults. Another limitation of the study is that the data reported use of various smoking modes including heated tobacco products, research to examine use of ENDS, which is becoming increasingly popular in the community, is warranted.

## 5 Conclusion

The study reported tobacco smoking prevalence, by methods of use, among adult population in Abu Dhabi, UAE. It examined the smoking prevalence during pre-, peri- and post-COVID-19 time periods. Results highlight a general decrease in prevalence levels when compared to previous studies, however an increase in use of heated tobacco products (Midwakh) was noted. There was a significant decline in the prevalence rates from the pre-COVID to peri-COVID period, that persisted to post-COVID era through 2023. Males and UAE nationals had higher smoking rates compared to females and non-nationals. Future efforts may address surveillance of use of new and emerging products such as ENDS and focus on designing interventions to sustain the drop in smoking levels brought by the pandemic, thus regulating the levels before they start to resurge. Furthermore, premarital screening clinics can serve as a significant, potential venue to educate the young adult population about potential risk of all tobacco use on self, family and future offsprings as well as provide counseling and referral for tobacco cessation program.

## Data Availability

The raw data supporting the conclusions of this article will be made available by the authors, without undue reservation.
