# Supplementary material for: Tobacco smoking patterns in the emirate of Abu Dhabi, United Arab Emirates: a comprehensive analysis of trends before and after the onset of the COVID-19 pandemic
Source: Front Public Health. 2025 Jun 26;13:1607965. doi: 10.3389/fpubh.2025.1607965 (PMC12241038; doi:10.3389/fpubh.2025.1607965)
Supplement: Supplementary file 1 [file Data_Sheet_1.docx]

**Supplementary Material**

**S1 Table:** Annual adult smoking prevalence by type based on gender and nationality status, Abu Dhabi 2019-23 (premarital screening program applicants) 

| **Type Tobacco** | | **National** | | | | | **Expatriate** | | **Overall** | | |
| --- | --- | --- | --- | --- | --- | --- | --- | --- | --- | --- | --- |
|  | *Female*  *N* | | *Male*  *N* | | | *Female*  *N* | | *Male*  *N* | *Female*  *N (%)* | *Male*  *N (%)* | *Overall*  *N (%)* |
| **Pre-COVID 2019 (N=15333)** | | | | | | | | | | | |
| **Cigarette** | 12 (0.3) | | 596 (11.0) | | | 153 (4.8) | | 574 (21.6) | 165 (2.3) | 1170 (14.5) | 1335 (8.7) |
| **Midwakh** | 9 (0.2) | | 976 (18.0) | | | 6 (0.2) | | 96 (3.6) | 15 (0.2) | 1072 (13.2) | 1087 (7.1) |
| **Waterpipe** | 18 (0.4) | | 258 (4.8) | | | 88 (2.8) | | 147 (5.5) | 106 (1.4) | 405 (5.0) | 511 (3.3) |
| **Overall** | 39 (1.0) | | 1830 (33.7) | | | 247 (7.8) | | 817 (30.7) | 286 (3.9) | 2647 (32.7) | 2933 (19.1) |
| **Peri-COVID 2020 (N=18814)** | | | | | | | | | | | |
| **Cigarette** | 11 (0.2) | | 724 (10.0) | | | 94 (3.0) | | 440 (16.4) | 105 (1.2) | 1164 (11.7) | 1269 (6.7) |
| **Midwakh** | 8 (0.1) | | 1312 (18.0) | | | 6 (0.2) | | 121(4.5) | 14 (0.2) | 1433 (14.4) | 1447 (7.7) |
| **Waterpipe** | 10 (0.2) | | 216 (3.0) | | | 57 (1.8) | | 79 (3.0) | 67 (0.8) | 295 (3.0) | 362 (1.9) |
| **Overall** | 29 (0.5) | | 2252 (31.0) | | | 157 (5.0) | | 640 (24.0) | 186 (2.2) | 2892 (29.1) | 3078 (16.4) |
| **2021 (N=15626)** | | | | | | | | | | | |
| **Cigarette** | 10 (0.2) | | 596 (9.7) | | | 31 (1.2) | | 275 (13.1) | 41 (0.6) | 871 (10.5) | 912 (5.8) |
| **Midwakh** | 3 (0.06) | | 839 (13.6) | | | 1 (0.03) | | 82 (3.9) | 4 (0.1) | 921 (11.2) | 925 (5.9) |
| **Waterpipe** | 8 (0.2) | | 175 (2.8) | | | 33 (1.3) | | 67 (3.2) | 41 (0.6) | 242 (2.9) | 283 (1.8) |
| **Overall** | 21 (0.4) | | 1610 (26.2) | | | 65 (2.6) | | 424 (20.2) | 86 (1.2) | 2034 (24.6) | 2120 (13.6) |
| **Post-COVID 2022 (N=13034)** | | | | | | | | | | | |
| **Cigarette** | | 2 (0.05) | | 503 (9.9) | 36 (1.6) | | | 210 (11.3) | 38 (0.6) | 713 (10.3) | 751 (5.8) |
| **Midwakh** | | 4 (0.1) | | 660 (13.0) | 6 (0.3) | | | 50 (2.7) | 10 (0.2) | 710 (10.2) | 720 (5.5) |
| **Waterpipe** | | 6 (0.2) | | 173 (3.4 | 19 (0.9) | | | 58 (3.1) | 25 (0.4) | 231 (3.3) | 256 (2.0) |
| **Overall** | | 12 (0.3) | | 1336 (26.3) | 61 (2.8) | | | 318 (17.1) | 73 (1.2) | 1654 (23.9) | 1727 (13.2) |
| **2023 (N=11984)** | | | | | | | | | | | |
| **Cigarette** | | 0 (0) | | 449 (9.2) | 20 (1.1) | | | 128 (8.1) | 20 (0.3) | 647 (10.1) | 667 (5.6) |
| **Midwakh** | | 1 (0.03) | | 661 (13.6) | 2 (0.01) | | | 61 (3.9) | 3 (0.1) | 722 (11.2) | 725 (6.0) |
| **Waterpipe** | | 6 (0.2) | | 167 (3.4) | 25 (0.1) | | | 61 (3.9) | 31 (0.6) | 228 (3.5) | 259 (2.2) |
| **Overall** | | 7 (0.2) | | 1277 (26.2) | 47 (2.6) | | | 250 (16.0) | 54 (1.0) | 1597 (24.8) | 1651 (13.8) |

**S2 Figure**: Overall trend in smoking in the five year period (2019-2023) among male participants of the pre-marital screening program based on smoking type


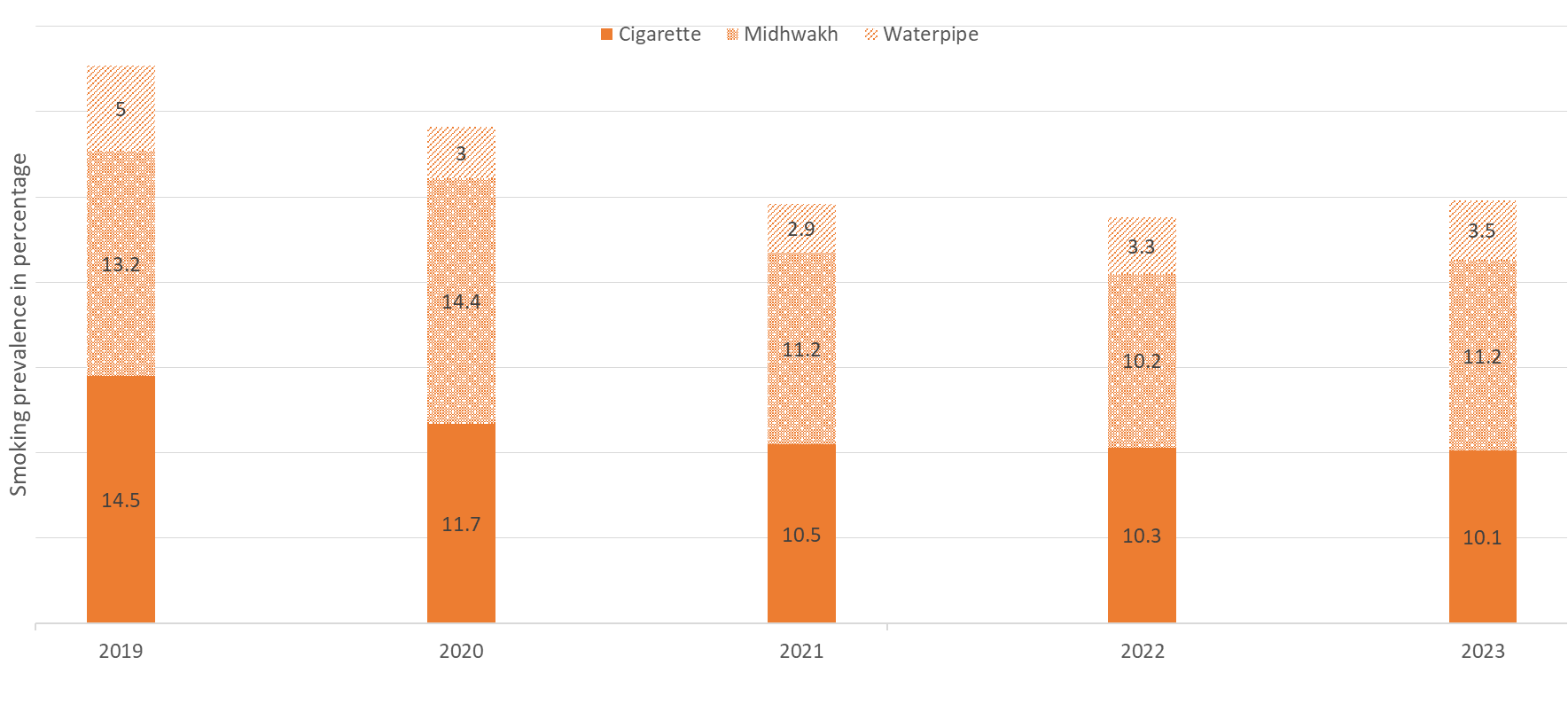


**S3 Figure**: Smoking prevalence across five years (2019-2024) among male participants of the pre-marital screening program based on smoking type
